# Sodium Reduction Through Sensory Interactions With NaCl: Strategies and Underlying Mechanisms

**DOI:** 10.1002/fsn3.70548

**Published:** 2025-07-04

**Authors:** Xiaohan Li, Bolin Shi, Rui Chen, Hehe Li, Lulu Zhang, Lei Zhao

**Affiliations:** ^1^ Department of Food Science and Engineering, College of Biological Sciences and Technology, Beijing Key Laboratory of Forest Food Processing and Safety, Hebei Province Key Laboratory of Sustainable Utilization and Development of Forest Food Resources Beijing Forestry University Beijing China; ^2^ Food and Agriculture Standardization Institute, Key Laboratory of Food Sensory Analysis, State Administration for Market Regulation China National Institute of Standardization Beijing China; ^3^ School of Food and Health Beijing Technology and Business University Beijing China

**Keywords:** chemesthesis, NaCl, saltiness enhancement, sensory interaction, sodium reduction

## Abstract

Excessive sodium intake from processed foods poses significant health risks, necessitating effective sodium‐reduction strategies in the food industry. This review explores sensory interactions between sodium chloride and flavor stimuli, including taste (sweetness, sourness, bitterness, and umami), orthonasal and retronasal olfaction (e.g., soy sauce, herbs, and spices), and chemesthetic sensations (e.g., tingling and burning), to enhance saltiness perception while reducing sodium content. Sensory interactions, particularly with retronasal olfaction and chemesthesis, can achieve sodium reductions of up to 75% and 39%, respectively, in various food matrices. Mechanistic insights reveal that top‐down regulation by higher neural centers, such as the insular and orbitofrontal cortex, enhances saltiness perception through peripheral and central nervous system pathways. These findings provide a practical foundation for the food industry to develop sodium‐reduction products.

## Graphical abstract

We proposed effective sodium‐reduction strategies by analyzing the mechanisms of sensory interactions between sodium chloride and flavor stimuli at peripheral and central nervous system levels. The results indicate that top‐down regulation by higher neural centers (e.g., insular and orbitofrontal cortex) plays a key role in enhancing saltiness perception.

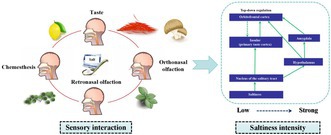

## 1 Introduction

Sodium chloride (NaCl), commonly known as salt, is a widely used condiment in daily life. Sodium, a key component of NaCl, is an essential nutrient that supports nerve and muscle function, signal transmission, and other physiological processes (Rosa, Pinna, et al. 2022). NaCl imparts saltiness to food, reduces bitterness, stimulates appetite, and elicits other sensory hedonic effects. It also enhances the physical and chemical properties of food, such as water retention, rheological characteristics, and microbial stability (Gong et al. 2022; Bower et al. 2018). However, excessive NaCl intake poses significant health risks, contributing to cardiovascular diseases, stroke, gastritis, and immune system disorders. Therefore, reducing NaCl consumption is crucial for promoting global health (Hunter et al. 2022). The World Health Organization and the US National Academy of Sciences recommended daily salt intake limits of 5 and 5.85 g for adults, respectively (Hunter et al. 2022). Despite these recommendations, the global average daily salt intake is approximately 10.0 g, with most countries exceeding recommended limits (Hendriksen et al. 2015; Trieu et al. 2015; Afshin et al. 2019; Zhang, Zhao, Gao, et al. 2020; Zhang, Zhao, Zhang, et al. 2020; Tsuchihashi 2022; Suzuki et al. 2023). For instance, in China, the average daily salt intake is 12.0 g, with over 90% of adults exceeding the recommended standard (Fang et al. 2020). Only 7.3% of 7665 study participants accurately identified the salt intake level recommended in the Chinese Dietary Guidelines (Han et al. 2022). Furthermore, most daily salt intake (70%–75%) originates from processed foods, with 10%–15% each from natural foods and salt added during cooking (Luan et al. 2020). Among processed foods, broths, sauces, and spreads have the highest sodium content, up to 27,105 mg per 100 g (Webster et al. 2010; Gong et al. 2022). Thus, sodium reduction remains a challenging yet crucial goal for the food processing industry.

Several developed countries, including Finland, the United Kingdom, France, the United States, Canada, and Japan, have successfully implemented sodium‐reduction initiatives, achieving notable reductions in residents' salt consumption despite still exceeding recommended levels (Tsuchihashi 2022). In response, the Chinese government has set a target to reduce average daily NaCl intake by 20% by 2030, as outlined in the “Healthy China 2030” plan (Zhang, Zhao, Zhang, et al. 2020). Local governments have also launched sodium‐reduction initiatives in various provinces and cities (Chen et al. 2020; Han et al. 2022). In 2018, the Salt Reduction Guide for China's Food Industry was published, proposing a phased 20% reduction in mean sodium content by 2030. In 2019, the Chinese Nutrition Society developed the “10 Core Information on Salt Reduction for Chinese Residents”, encouraging the public to limit salt intake to 6 g per day initially and achieve the “Healthy China Action” goal of 5 g per day by 2030. Although government mandates promote reduced sodium in food products, lower sodium content may affect product acceptability. Developing sustainable and effective sodium‐reduction strategies is essential to mitigate health risks from excessive sodium intake while preserving dietary patterns. This review provides a comprehensive overview of sodium‐reduction strategies, exploring sensory interactions, such as saltiness paired with other basic tastes, orthonasal and retronasal olfaction, and chemesthetic sensations, to enhance saltiness perception while reducing sodium content, and examines the mechanisms underlying these enhancements.

## 2 Overview of Sodium‐Reduction Strategies

The perception of saltiness in food is influenced by factors such as sodium release within the food matrix, sodium ion diffusion in the oral cavity, and taste receptor sensitivity. Numerous sodium‐reduction strategies have been implemented in food applications, yielding promising results. This section outlines effective strategies for reducing sodium content.

### 2.1 NaCl Substitutes

One method for reducing sodium intake is partially replacing NaCl with substitutes. Common NaCl substitutes, such as potassium chloride, potassium lactate, potassium citrate, calcium chloride, calcium lactate, calcium ascorbate, and magnesium chloride, are used (Inguglia et al. 2017; Wang et al. 2021). Ideally, substitutes should elicit saltiness, but compounds like potassium chloride may impart bitter, astringent, or metallic taste, limiting their use in food production (Van Buren et al. 2016). Previous research has shown that some amino acids (e.g., L‐glutamate and L‐arginine), related peptides, and arginyl dipeptides (e.g., Arg‐Pro, Arg‐Ala, and Ala‐Arg) exhibit saltiness‐enhancing functions (Xu et al. 2017; Chen et al. 2021).

### 2.2 Strategies for Reducing NaCl Based on Food Colloids

Emulsion‐based NaCl delivery systems provide a practical approach to reducing sodium in semisolid and liquid foods. In an oil‐in‐water (O/W) system, Rietberg et al. (2012) found that the mass fraction of the aqueous phase, initial salt load, and surfactant concentration enhanced saltiness. However, ensuring emulsion stability throughout the shelf life of processed food while enabling rapid sodium release in the oral cavity requires further research. Torrico et al. (2015) found that saltiness enhancement was more pronounced with 20%–40% oil in a W/O system. Matos et al. (2013) demonstrated that quinoa starch modified with octylsuccinic anhydride encapsulated 1.6% NaCl in a W/O/W emulsion, maintaining over 90% encapsulation efficiency after 21 days. Chiu et al. (2015) prepared an OSA starch‐stabilized W/O/W emulsion that rapidly releases sodium under oral processing and saliva enzymes, achieving a maximum sodium reduction of 23.7%. Under the action of amylase and oral processing, the internal phase of OSA starch‐stabilized W/O/W emulsions demulsifies, releasing sodium to enhance saltiness. Studies suggest that low molecular weight emulsifiers, such as phospholipids and monoglyceryl esters, increase the fluidity and permeability of human intestinal Caco‐2 cell membranes (Wang et al. 2021). Since taste cell membranes also consist of phospholipid bilayers, these emulsifiers may promote sodium penetration and transport, thereby enhancing saltiness perception.

### 2.3 Flavor Enhancers

Flavor enhancers, such as guanosine disodium, sodium glutamate, yeast extract, certain amino acids, and Maillard reaction products, are tasteless substances that enhance saltiness perception by activating gustatory receptors in the oral cavity (Mukeshimana et al. 2019; Wang et al. 2021). Gao et al. (2022) found that flavor compounds from Chinese Douchi, including 2‐ethyl‐3,5‐dimethyl pyrazine, 2,5‐dimethyl pyrazine, and dimethyl trisulfide, significantly enhanced saltiness perception in NaCl solutions. Fermented condiments like soy sauce, shrimp oil, fish sauce, and shrimp paste are popular in China and Southeast Asia for their unique flavor profiles. However, these condiments often contain high‐sodium levels. Desalination techniques, such as electrodialysis, nanofiltration, and rotary evaporation, may reduce sodium but can diminish popular flavors (Chindapan et al. 2018). Overall, amino acids, yeast extract, and other flavor enhancers can interact with saltiness to enable sodium reduction.

### 2.4 Particle Size Reduction Method for NaCl Crystals

While NaCl substitutes, food colloids, and flavor enhancers have proven effective in regulating sodium levels, the particle size reduction method for NaCl crystals has been successful in solid foods. Quilaqueo and Aguilera (2016) prepared NaCl crystals with smaller grain sizes using spray drying, anti‐solvent crystallization, and mechanical grinding, and applied them to salty snacks (e.g., French fries, potato chips, and biscuits). They found that NaCl particle size was reduced by three orders of magnitude compared to standard commercial salt, decreasing the amount needed for cheese crackers by 25%–50%. Vinitha et al. (2021) used electrohydrodynamic atomized drying to produce nano‐sized (520 nm) NaCl, achieving a 65.34% sodium reduction while maintaining similar saltiness levels.

## 3 Overview of Sensory Interactions Between NaCl and Other Flavor Stimuli

**FIGURE 1:**
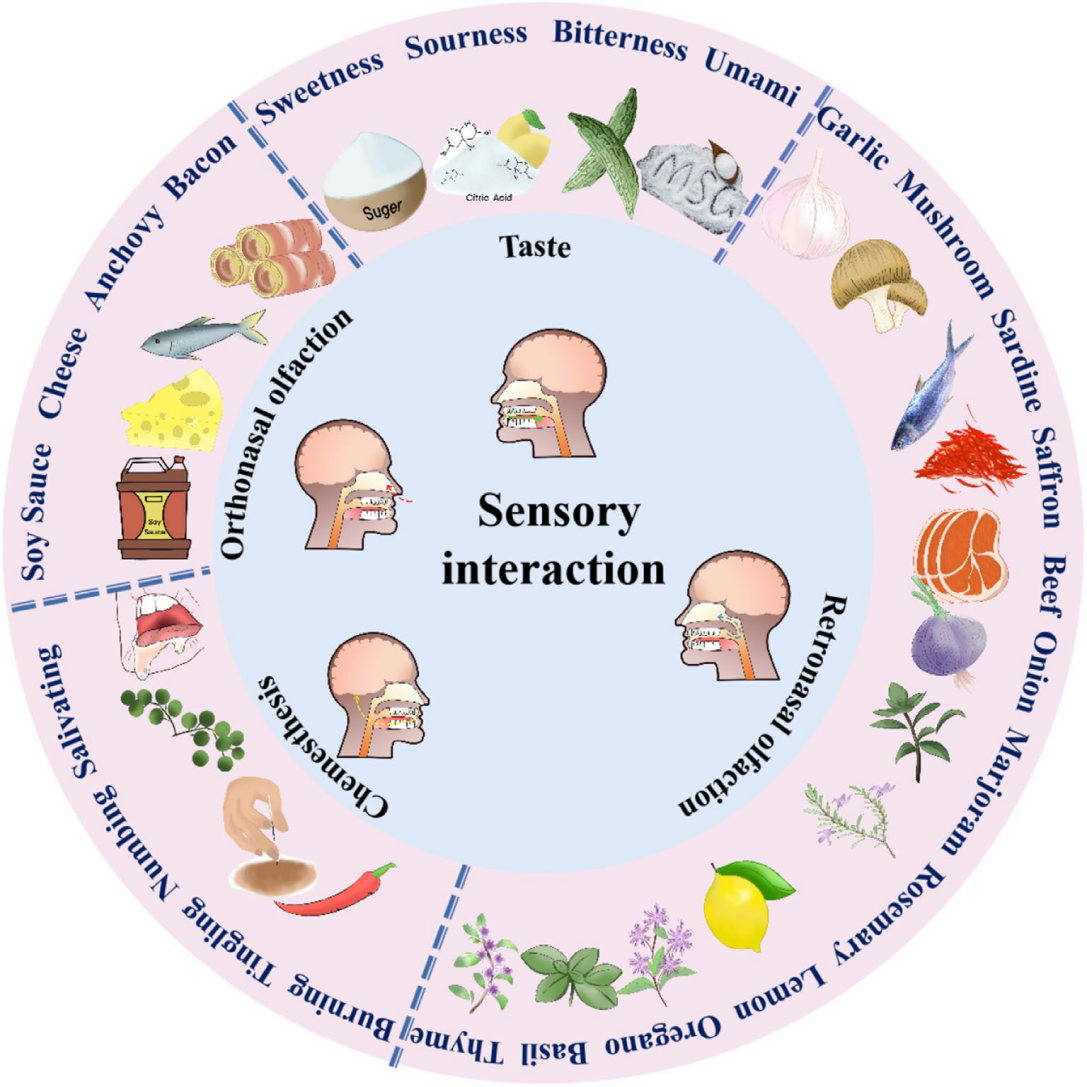
Saltiness enhancement through sensory interactions between NaCl and flavor stimuli, including basic tastes (sweetness, sourness, bitterness, and umami), orthonasal olfaction, retronasal olfaction, and chemesthesis.

Although many salt‐reduction strategies have been reported, sensory interactions between NaCl and flavor stimuli have rarely been summarized. Flavor is the sensory impression of food, primarily determined by taste, olfaction, and chemesthesis (Tremblay and Frasnelli 2018; Moss et al. 2023). This review summarizes sensory interactions between NaCl and other taste stimuli, orthonasal olfaction, retronasal olfaction, and chemesthesis. Orthonasal olfaction refers to odor perception via the nasal cavity during inhalation, primarily detecting volatile compounds. Retronasal olfaction involves odor perception during exhalation, as volatile compounds from food or beverages travel through the nasopharynx to olfactory receptors, significantly contributing to flavor. Chemesthesis is the sensory response triggered by chemical stimuli interacting with the trigeminal nerve, producing sensations such as burning, tingling, or irritation, distinct from taste or smell. As shown in Figure 1, taste stimuli (sweetness, sourness, bitterness, and umami), orthonasal olfaction (soy sauce, cheese, anchovy, and bacon), retronasal olfaction (mushroom, soy sauce, saffron, beef, and spices and herbs), and chemesthetic sensations (tingling, numbing, and burning) all play key roles in saltiness enhancement.

### 3.1 NaCl and Other Taste Stimuli

Numerous studies have reported sensory interactions between NaCl and basic taste stimuli (Figure 1 and Table 1). Research shows that detection thresholds and supraliminal intensities are unrelated, reflecting different levels of human perception (Zhang et al. 2023). At the peri‐threshold level, the saltiness threshold of NaCl decreased by 60%, 56%, and 33% in NaCl‐sucrose, NaCl‐citric acid, and NaCl‐quinine hydrochloride binary solutions, respectively (Stevens 1995). Hatae et al. (2009) reported that adding vinegar to NaCl solution significantly lowered salt detection and recognition thresholds. At the suprathreshold level, mixing NaCl with other taste qualities results in enhancement, suppression, or no effect (Keast and Breslin 2003). Adding umami‐evoking ingredients, such as monosodium glutamate, disodium succinate, 5′‐inosinate disodium, and nucleotide disodium, is a sodium‐reduction strategy, with medium‐concentration disodium succinate achieving a maximum sodium reduction of 24.25% (Sun et al. 2022).

**TABLE 1:** Representative findings on sodium reduction through sensory interactions between NaCl and basic tastes (sweetness, sourness, bitterness, and umami), orthonasal olfaction, retronasal olfaction, and chemesthesis.

Sensory dimension	Sensory interactive	Participants	Sensory evaluation method	Food matrix	Saltiness enhancement effect	References
Other taste	Saltiness and sweetness	5 males and 6 females with ages between 18 and 27 years	Forced‐choice method	Aqueous solution containing 0.128 M NaCl and 0.40 M sucrose.	Saltiness threshold in NaCl‐sucrose binary solution decreased by 60%.	Stevens (1995)
	Saltiness and sourness	5 males and 6 females with ages between 18 and 27 years	Forced‐choice method	Aqueous solution containing 0.128 M NaCl and 0.0042 M citric acid.	Saltiness threshold in NaCl‐citric acid binary solution decreased by 56%.	Stevens (1995)
	Saltiness and sourness	40 females with ages between 20 and 22 years	Paired difference test	Aqueous solution containing NaCl (1.280 × 10^−1^ to 3.05 × 10^−8^ M), rice vinegar (5.12 × 10^−3^ to 1.221 × 10^−9^ M), and black rice vinegar (5.12 × 10^−3^ to 1.221 × 10^−9^ M).	Adding vinegar to the NaCl solution significantly lowered saltiness detection and recognition thresholds.	Hatae et al. (2009)
	Saltiness and bitterness	5 males and 6 females with ages between 18 and 27 years	Forced‐choice method	Aqueous solutions containing 0.128 M NaCl and 0.00025 M quinine hydrochloride.	The saltiness threshold of NaCl in a binary solution of NaCl‐quinine hydrochloride decreased by 33%.	Stevens (1995)
	Saltiness and umami	6 males and 6 females (mean age = 20.3 years)	Visual analog scale (VAS)	Aqueous solution containing NaCl (0%, 0.18%, 0.58%, and 0.80%) and 0.10% monosodium glutamate (MSG).	MSG enhanced saltiness intensity. The peak intensity and AUC of the TI saltiness curve was enhanced by MSG addition.	Onuma et al. (2018)
	Saltiness and umami	4 males and 12 females (mean age = 31.3 ± 4.7 years)	Generalized Labeled Magnitude Scale (gLMS)	Aqueous solution containing varing concentrations of NaCl, MSG, disodium succinate (WSA), 5′‐inosinate disodium (IMP), and nucleotide disodium (I + G).	Umami carriers (WSA, MSG, IMP, and I + G) effectively enhanced saltiness in NaCl solutions at varying concentrations. WSA at moderate intensity achieved a sodium reduction of 24.25%.	Sun et al. (2022)
Orthonasal olfaction	Saltiness and soy sauce	13 females with ages between 17 and 23 years	21‐point scale	Aqueous solution containing: NaCl (0, 0.056 [weak] and 0.32 M [strong]) and commercially available soy sauce.	Solutions with zero (water) and weak NaCl concentrations were perceived as saltier with soy sauce than without.	Djordjevic et al. (2004)
	Saltiness and cheese	5 males and 3 females with ages between 23 and 51 years	10 cm unstructured horizontal scale	Model cheese containing NaCl (44–1400 ppm) and cheese aroma compounds.	Cheese aroma significantly enhanced saltiness perception with a nose‐clip than without a nose‐clip.	Pionnier et al. (2004)
	Saltiness and bacon	19 females and 6 males (mean age ± SD = 25 ± 4 years)	VAS	NaCl concentrations ranged between 0.16 and 0.64 M. Bacon odor.	Bacon odor enhanced saltiness in low‐concentration NaCl solutions more than in high‐concentration solutions.	Seo et al. (2013)
	Saltiness and soy sauce	6 males and 6 females (mean age = 20.3 years)	VAS	NaCl concentrations ranged between 0% (water) and 0.80%. Soy sauce.	Peak intensity and AUC of time intensity curve were enhanced by soy sauce addition.	Onuma et al. (2018)
Retronasal olfaction	Saltiness and spice blends	34 females and 26 males with ages between 22 and 56 years	Saltiness threshold measurements, and consumer acceptability test	Vegetable soup containing 0.93% salt or 0.45% salt equivalent. 0.15% rosemary, 0.1% lactoferrin hydrolysate, and 0.05% spice blend.	The addition of rosemary, lactoferrin hydrolysate, or spice blend to reduced sodium soups allowed for a salt reduction of 48%.	Mitchell et al. (2013)
	Saltiness and spice blends	62 untrained panelists	9‐point hedonic scale	Margarine with reduced sodium content varying from 0 to 100%. Mixture A contained green onion, garlic, marjoram, and thyme. Mixture B contained lemon, oregano, basil, and thyme.	The addition of spice mixture A and spice mixture B resulted in NaCl reduction of 75% and 50%, respectively.	Lopes et al. (2014)

	Saltiness and herb and spice blends	A trained sensory panel	15 cm unstructured line with scales from 0 to 100	Standard tomato soup. 0.5% (w/w) salt content and three different samples with reduced salt contents. Mixture of basil (added basil, black pepper, celery, and garlic), cumin and coriander (added cumin, coriander, ground celery seed, and garlic), and oregano modification (added oregano, bay leaves, garlic, celery, and black pepper).	Herb and spice blends enhanced saltiness perception and compensate for 53% salt reduction.	Ghawi et al. (2014)
	Saltiness and garlic	44 hypertensive subjects	Preference test	Rice porridge prepared with 100 g rice and 600 mL water. Salt concentration: 0.25%, 0.50%, and 0.75%. Garlic concentration: 1.4 g/100 g porridge.	31.8% of the treatment group preferred food samples with lower salt content when garlic was added.	Nugrahani and Afifah (2016)
	Saltiness and herb extracts	29 males and 51 females with ages between 19 and 24 years	VAS	Water solution containing NaCl and herb extracts (basil, parsley, oregano, anise, and rosemary).	The addition of 0.35% herb extracts enhanced the saltiness intensity of NaCl by 1.13–1.22 times, and reduced the NaCl by 10%–20%.	Kohri et al. (2020)
	Saltiness and soy sauce	75 females with ages between 21 and 26 years	Paired comparison test	Soup containing NaCl, and buckwheat noodle soup with cooked (uncooked) soy sauce and 3‐Me‐BuOH added.	3‐Methyl‐1‐butanol (3‐Me‐BuOH) enhanced saltiness through the retronasal odor of soy sauce, compensating for a 6% NaCl reduction.	Manabe et al. (2020)
Saltiness and beef flavor	15 females and 6 males with ages between 18 and 30 years	Rating	Green‐pea soup containing NaCl, with PP (50% base solution + 50% Evian water), PPS1 (50% base solution + 25% salt solution + 25% Evian water), PPS1B (50% base solution + 25% salt solution + 25% aromatic solution), or PPS2 (50% base solution + 50% salt solution) added.	All salt‐added solutions (PPS1, PPS1B, and PPS2) were perceived as saltier than control solutions (PP).	Sinding et al. (2021)
Chemesthesis	Saltiness and burning	9 males and 18 females (mean age = 21.3 ± 2.4 years)	Paired difference test	NaCl containing capsaicin solutions.	Stronger saltiness perception was observed when adding 0.5 or 1 μM capsaicin to a 75 mM NaCl solution.	Narukawa et al. (2011)
	Saltiness and tingling	31 young adults (15 males and 16 females, mean age = 23.4 ± 3.0 years) and 29 old participants (15 males and 14 females, mean age = 66.2 ± 4.2 years)	15 cm linear scale	NaCl solutions prepared with spring water and slightly pungent solutions.	NaCl content was reduced by 34.4% in the younger group and 4.4% in the older group.	Zhang, Zhao, Zhang, et al. (2020)
	Saltiness and tingling	34 males and 34 females (mean age = 20.4 ± 2.0 years)	gLMS	NaCl solution contained Sichuan pepper oleresin.	The low pungency solution enhanced saltiness perception, and the maximum percent reduction of NaCl was 39.06%.	Zhang, Zhao, Gao, et al. (2020)
	Saltiness and tingling and burning	6 males and 9 females with ages between 20 and 38 years	gLMS	NaCl solutions. Capsaicin solutions. Sichuan pepper oleoresin solutions. C + P mixture containing capsaicin and Sichuan pepper oleoresin at various combinations.	C + P significantly enhanced saltiness of the solutions containing NaCl, and the maximum salt reduction were 37.65%.	Wang et al. (2022)
	Saltiness and burning	45 males and 55 females with ages between 18 and 65 years	gLMS	A commercially available soup (e.g., piperine and salt)	Saltiness intensity was perceived as significantly higher in the piperine soup than the control.	Moss et al. (2023)

### 3.2 NaCl and Orthonasal Olfaction Stimuli

Odor‐induced taste enhancement (OITE) results from the integration of taste and odor with taste perception (Syarifuddin et al. 2016). Only consistent and familiar flavor mixtures produce OITE efficiently. For example, Djordjevic et al. (2004) reported that salty‐congruent soy sauce odor enhanced perceived saltiness. Pionnier et al. (2004) explored the effect of propionic acid, butyric acid, and diacetyl on the saltiness intensity of cheese and found that saltiness intensity was higher without a nose‐clip, indicating that congruent aromas enhance taste perception. Seo et al. (2013) found that a salty‐congruent odor produces significantly higher neural activation in brain regions associated with odor‐taste integration (e.g., the insular, anterior cingulate cortex, and orbitofrontal cortex) than an incongruent odor. Low‐concentration odorants enhance saltiness perception more effectively than high‐concentration odorants. Therefore, incorporating low‐concentration orthonasal olfactory odorants can compensate for reduced NaCl levels in food by amplifying saltiness perception (Figure 1 and Table 1). Conversely, odors unrelated to NaCl, such as carrot aroma, may decrease saltiness ratings in low‐salt solutions.

### 3.3 NaCl and Retronasal Olfaction Stimuli

**FIGURE 2:**
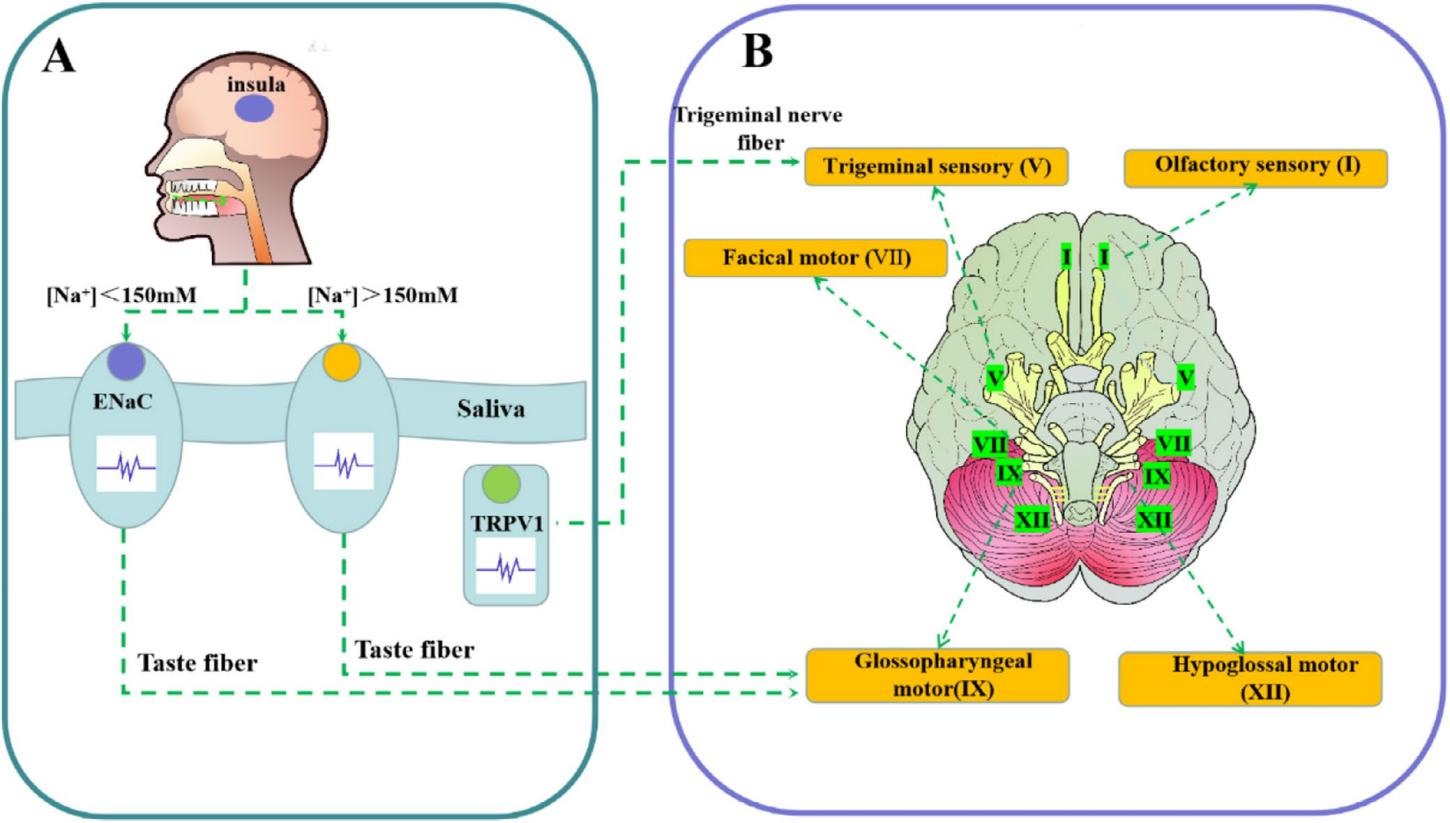
Mechanisms of NaCl saltiness perception at the peripheral nervous system level (A) and central nervous system level (B).

Retronasal olfaction significantly enhances saltiness in various flavor combinations (Table 1 and Figure 2). Manabe et al. (2020) showed that 3‐methyl‐1‐butanol enhances saltiness through the retronasal odor of soy sauce. Rosa, Loy, et al. (2022) demonstrated that aromatic herbs and spices significantly enhance saltiness perception in patients with hyposmia; Rosa, Pinna, et al. (2022) also found that sea salt flavored with orange fruit and saffron most effectively enhanced saltiness perception. Herb and spice blends, including rosemary and lactoferrin hydrolysate, significantly enhance saltiness (Manabe et al. 2020; Lopes et al. 2014; Ghawi et al. 2014; Nugrahani and Afifah 2016; Kohri et al. 2020; Figure 2). Reformulating soups with herbs and spices and repeated exposure enhanced saltiness intensity, achieving a maximum salt reduction of 22.8% (Ghawi et al. 2014). Adding spice mixture A (e.g., green onion, garlic, marjoram, and thyme) and spice mixture B (e.g., lemon, oregano, basil, and thyme) reduced sodium by up to 75% and 50%, respectively (Lopes et al. 2014). Nugrahani and Afifah (2016) found that 31.8% of the treatment group preferred lower sodium rice samples (0.25% and 0.05%) with added garlic, compared to 18.2% in the control group. Kohri et al. (2020) quantified that 0.35% herb extracts enhance NaCl saltiness by 1.13–1.22 times, potentially reducing sodium usage by 10%–20%. Thus, we hypothesize that carefully selected odors can compensate for NaCl reduction in food through sensory interactions between retronasal olfaction and saltiness (Figure 2).

### 3.4 NaCl and Chemesthetic Stimuli

An increasing number of studies investigate saltiness enhancement through sensory interactions between chemesthetic stimuli and NaCl (Narukawa et al. 2011; Zhang, Zhao, Gao, et al. 2020; Zhang, Zhao, Zhang, et al. 2020; Wang et al. 2022; Moss et al. 2023; Table 1 and Figure 2). Narukawa et al. (2011) found that adding 0.5 or 1 μM capsaicin enhanced human sensitivity to saltiness. Zhang, Zhao, Zhang, et al. (2020) systematically studied cross‐modal interactions between the tingling sensation evoked by Sichuan pepper and basic tastes, confirming that pungency from hydroxy‐sanshools in the aqueous phase enhances saltiness, allowing a potential sodium reduction of over 30% while preserving saltiness perception (Zhang, Zhao, Gao, et al. 2020). Wang et al. (2022) found that low and medium intensity burning and tingling mixtures enhanced saltiness perception, reducing NaCl by up to 26.09%. Recently, Moss et al. (2023) found that piperine significantly enhanced the saltiness intensity of a low‐sodium soup through cross‐modal interactions between pungency and saltiness.

Sensory interactions between NaCl and taste, orthonasal olfaction, retronasal olfaction, and chemesthesis effectively enhance saltiness perception. Retronasal olfaction stimuli significantly enhance saltiness through sensory interactions with NaCl. The highest sodium reduction, 75%, was achieved with spice blends in margarine (Lopes et al. 2014). Samples with common retronasal olfaction stimuli achieve sodium reductions exceeding 48% (Lopes et al. 2014; Ghawi et al. 2014; Mitchell et al. 2013). Chemesthesis stimuli, particularly tingling, yield a maximum sodium reduction of 39.06% (Zhang, Zhao, Gao, et al. 2020), with typical chemesthesis stimuli reducing sodium by over 30% (Zhang, Zhao, Zhang, et al. 2020; Wang et al. 2022). Umami (e.g., WSA) at moderate intensity achieves a sodium reduction of 24.25% (Sun et al. 2022). In contrast, orthonasal olfaction interactions have a less pronounced effect compared to retronasal olfaction (Pionnier et al. 2004; Seo et al. 2013). For example, Seo et al. (2013) reported that a NaCl solution with bacon odor was perceived as saltier than with odorless air, achieving a maximum sodium reduction of 13.60%. In summary, sensory interactions between flavor stimuli and NaCl offer a promising approach to amplify saltiness perception while reducing sodium content.

In addition to flavor quality, the concentration of specific stimuli significantly influences saltiness perception. For example, Zhang, Zhao, Gao, et al. (2020) found that pungency‐enhanced saltiness depends on NaCl concentration, pungent stimuli concentration, and individual sensitivity. Low pungency solutions notably increased perceived saltiness in hypersensitive and semisensitive groups, but not in hyposensitive groups. Similarly, low‐concentration odorants elicit greater saltiness enhancement than high‐concentration odorants (Seo et al. 2013). Moreover, saltiness enhancement is more pronounced at low to moderate NaCl concentrations than at high concentrations (Zhang, Zhao, Gao, et al. 2020; Zhang, Zhao, Zhang, et al. 2020; Hatae et al. 2009; Wang et al. 2022; Moss et al. 2023).

These sensory interactions provide significant potential for sodium reduction, yet their application in complex food systems remains challenging. First, much research has been conducted in simplified matrices (e.g., aqueous solutions, model cheeses, soups, margarine, and rice porridge), which may not fully reflect complex food matrices (Table 1). Saltiness perception in these matrices may differ from that in real foods, necessitating further investigation in real food systems. Second, dietary habits and preferences for flavor stimuli influence saltiness perception (Taladrid et al. 2020). For example, Reinbach et al. (2010) noted that spicy food consumption can reduce cravings for salty foods. Ghawi et al. (2014) found that reducing NaCl in tomato soup with added spices could decrease consumer preference. Li et al. (2017) showed that new flavor stimuli can reduce preference for saltiness, promoting sodium reduction. Third, many studies involve young participants, whose taste thresholds are lower than those of older individuals (over 65 years) (Fukunaga et al. 2005). Age‐related declines in taste perception, including reduced taste bud density after age 50, decrease saltiness sensitivity (Piochi et al. 2019). Psychophysical testing reveals significant taste function decline with age, due to degradation of gustatory peripheral tissues, neural signatures in the central nervous system, and altered cerebellar‐cortical connectivity (Iannilli et al. 2017; Piochi et al. 2019). Since excessive NaCl intake poses greater health risks for middle‐aged and older populations, evaluating saltiness enhancement in these groups is critical. In conclusion, while sensory interaction strategies offer promising prospects for sodium reduction, addressing complexities such as food matrices, dietary preferences, and age‐related variations is essential to advance this field and effectively tackle excessive salt intake.

## 4 Saltiness Perception Mechanisms

### 4.1 Peripheral Nervous System Level

Saltiness is primarily induced by NaCl, KCl, CaCl_2_, NH_4_Cl, amino acids, and other substances that stimulate salt receptors on the tongue, glossopharynx, and larynx (Avery 2021). Physiological studies in mammals have identified three receptor transduction pathways for saltiness perception (Figure 2A): (1) The amiloride‐sensitive (AS) pathway, mediated by the epithelial sodium channel (ENaC), which is highly selective for sodium below 150 mM (Bigiani 2020). However, the specific cell types mediating the AS pathway remain unclear. (2) The amiloride‐insensitive (AI) pathway, with unknown sodium receptor candidates, is more sensitive to non‐sodium salty components than to sodium alone (Bigiani 2020). The AI pathway is mediated by type III cells for sour taste (Teng et al. 2019) and type II cells for bitter taste (Finger and Barlow 2021; Roebber et al. 2019). (3) The transient receptor potential vanilloid 1 (TRPV1) pathway, an ion channel expressed by trigeminal nerve endings, mediates saltiness perception. Like AI taste cells, trigeminal fibers likely detect non‐sodium salty components rather than sodium alone (Simon and Gutierrez 2017).

In murine fungiform papillae, the AS pathway is preferentially activated at low NaCl concentrations, whereas the AI pathway is activated at high concentrations (Figure 2A). High‐sodium (> 150 mM) solutions can activate bitter taste receptors in type II cells, triggering intracellular calcium signaling and neurotransmitter release (Oka et al. 2013). Although AS, AI, and TRPV1 pathways are identified in rodents, their roles and interactions in human saltiness perception remain unclear. For example, the existence of ENaC‐mediated pathways in human taste buds is debated, and the role of trigeminal input through TRPV1 channels is not fully understood. Therefore, the precise mechanisms underlying saltiness transduction in humans require further investigation (Bigiani 2021).

### 4.2 Central Nervous System Level

**FIGURE 3:**
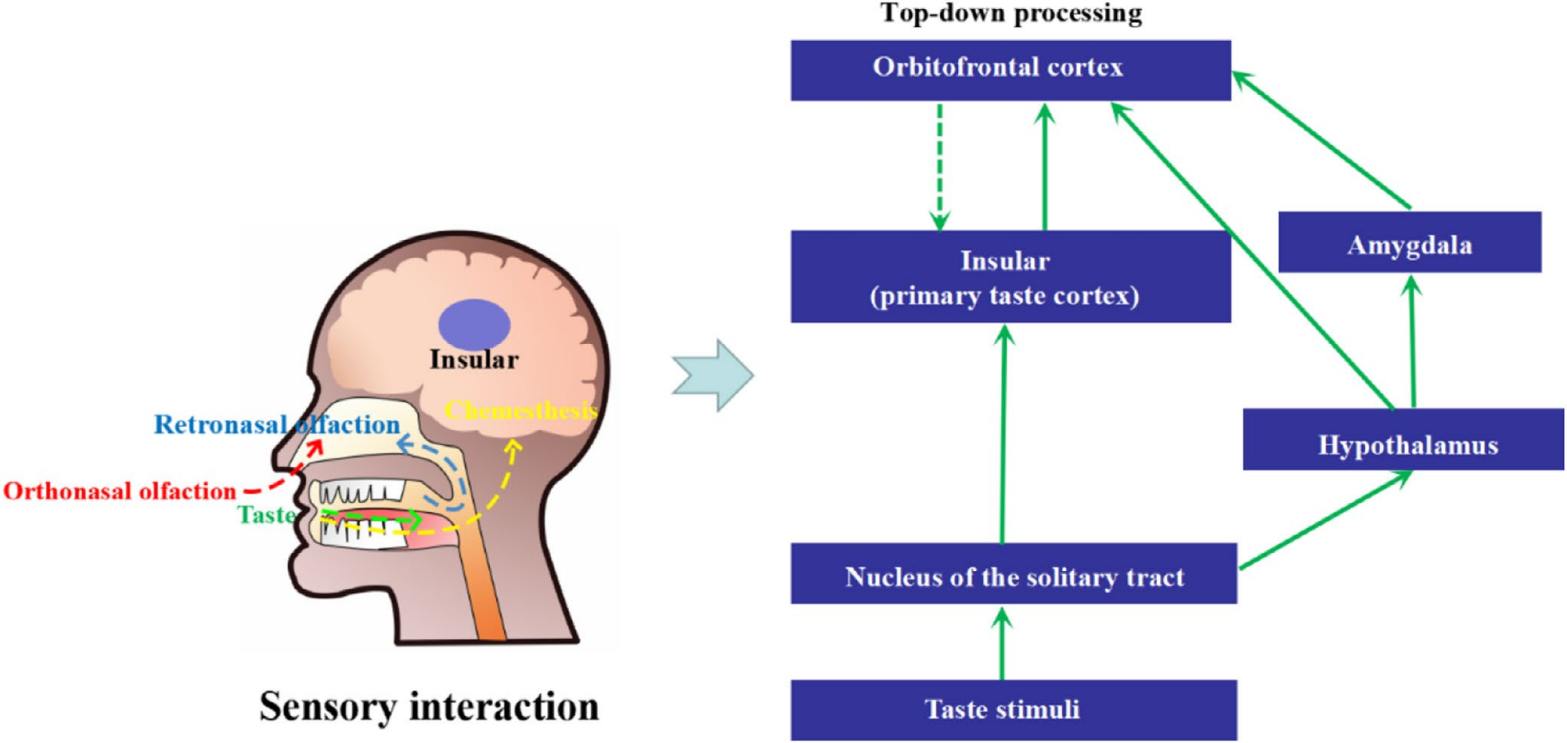
Mechanisms of saltiness enhancement through sensory interactions between NaCl and flavor stimuli in brain regions.

Brain regions involved in taste perception, primarily the right insular and cingulate gyrus, also contribute to emotional and somatosensory processing. Gustatory signals are encoded by the seventh, ninth, and tenth cranial nerves, which project information to the nucleus of the solitary tract in the medulla oblongata (Figure 2A,B). Gustatory signals are then transmitted from the nucleus of the solitary tract to higher brain regions via three pathways: (1) pons → lateral hypothalamus → amygdala → orbitofrontal cortex (OFC); (2) pons → insular (primary taste cortex) → OFC; and (3) pons → ventral posterior medial thalamus → somatosensory cortex (Su 2019; Figure 3). At the central nervous system level, the relationship between specific brain regions and responses to specific tastes requires further investigation. Avery (2021) proposed two hypotheses for taste perception: the topographic model and the population coding model. Studies in rodents, primates, and humans suggest that neuronal population encoding is a common taste representation pattern (Stettler and Axel 2009; Avery 2021).

In addition to population encoding, temporal encoding patterns in the brain are studied using electroencephalogram (EEG) techniques. One study showed that taste event‐related potentials can be recorded at 150–200 ms (Wallroth and Ohla 2018). The population coding model and temporal encoding patterns offer critical insights into neural mechanisms underlying sensory interactions.

## 5 Mechanisms of Saltiness Enhancement

Sensory interactions between NaCl and other flavor stimuli can occur at the peripheral and central nervous system levels (Keast and Breslin 2003). However, few studies have investigated the mechanisms of saltiness enhancement through sensory interactions. This review focuses on the mechanisms of saltiness enhancement by odor, burning, and tingling sensations.

### 5.1 Peripheral Nervous System Level

At the peripheral nervous system level, the mechanisms of saltiness enhancement by hydroxy‐sanshools and capsaicin have been studied. Lyall et al. (2004) found that 40 μM capsaicin stimulated the TRPV1 receptor while inhibiting the ENaC channel, significantly enhancing the saltiness signal in the chorda tympani nerve of mice. Li et al. (2017) found that 0.5 μM capsaicin significantly increased calcium signal amplitude in the tongues of freely moving mice. Using calcium imaging, Xu et al. (2019) confirmed that hydroxy‐sanshool‐related compounds enhanced NaCl sensitivity in type III cells of mice. Kapaun and Dando (2017) showed that burning sensations enhance sour taste and that calcitonin gene‐related peptide receptors on type III taste cells contribute to saltiness perception.

### 5.2 Central Nervous System Level

The central nervous system mechanisms of saltiness enhancement have been shown in Figure 3. Spatially, Li et al. (2017) confirmed that capsaicin and NaCl activation overlap in the insular, prefrontal cortex, and OFC. The metabolic activity of the insular, thalamus, and OFC depends on NaCl concentration. Capsaicin enhances and maintains this activity in the insular and OFC, even at reduced sodium concentrations, preserving saltiness perception.

Beyond the spatial perspective, a temporal viewpoint is essential for exploring saltiness enhancement mechanisms. Taste event‐related potentials can be recorded within 150–200 ms using electroencephalogram (EEG). Thus, EEG studies, with their high temporal resolution (measured in milliseconds), are critical for understanding these mechanisms. EEG studies show that consistent taste attributes elicit stable central response sites, event‐related potential (ERP) waveforms, and latencies (Hu and Zhang 2019). P1, N1, and P2 ERPs are generated at specific NaCl concentrations, with ERP amplitudes reflecting stimulus intensity; for example, 5 and 20 g/L NaCl produce P1N1 amplitudes at the Cz electrode of 19.7 ± 8.6 and 20.7 ± 10.5 μV, respectively. Sinding et al. (2021) showed that P1 amplitude remained unchanged, while P3 latency was prolonged, indicating that olfaction‐taste interactions occur in late‐stage processing in higher brain regions (e.g., OFC, peri‐insular, and dorsal insular). These findings suggest that top‐down regulation enhances saltiness perception in OITE. Sinding et al. (2021) hypothesized that top‐down regulation from higher neural centers enhances saltiness perception beyond primary region enhancement. However, further verification using imaging methods like fMRI or PET is needed to confirm neural conduction pathways for saltiness and tingling sensations. Top‐down regulation refers to cognitive and neural processes where higher brain regions (e.g., insular and OFC) modulate lower level sensory areas through attention, expectation, or memory, integrating multisensory inputs (e.g., olfactory or chemesthetic stimuli) to amplify saltiness perception at reduced sodium concentrations (Sinding et al. 2021; Keast and Breslin 2003; Small and Prescott 2005). Thus, sensory interactions, driven by central nervous system regulation, enhance saltiness perception.

## 6 Conclusion

We summarize effective sodium‐reduction strategies based on sensory interactions between NaCl saltiness and other flavor stimuli. Mechanistic insights reveal that top‐down regulation by higher neural centers (e.g., insular and OFC) enhances saltiness through peripheral and central nervous system pathways. Despite these advances, challenges remain in applying findings from simplified matrices to real foods, addressing age‐related variations in taste perception, and aligning sensory enhancements with consumer preferences. Future research should prioritize: (1) psychophysical studies to optimize flavor intensity and acceptability across diverse populations; (2) human‐based physiological studies to elucidate saltiness perception pathways; and (3) neuroimaging to map flavor‐induced neural responses. These efforts will promote the development of innovative, acceptable low‐sodium products, supporting global health initiatives to reduce excessive salt intake.

## Author Contributions

**Xiaohan Li:** writing – original draft (equal), writing – review and editing (equal). **Bolin Shi:** conceptualization (equal), methodology (equal), writing – review and editing (equal), writing – review and editing (equal). **Rui Chen:** methodology (equal), writing – review and editing (equal). **Hehe Li:** funding acquisition (equal), writing – review and editing (equal). **Lulu Zhang:** conceptualization (lead), formal analysis (lead), funding acquisition (lead), investigation (equal), methodology (equal), writing – original draft (lead), writing – review and editing (lead). **Lei Zhao:** conceptualization (equal), methodology (equal), supervision (equal), writing – review and editing (equal).

## Conflicts of Interest

The authors declare no conflicts of interest.

## Data Availability

No data was used for the research described in the article.
